# Beyond Otitis Media: When Mastoiditis Leads to Life‐Threatening Complications in Children

**DOI:** 10.1155/crpe/6697317

**Published:** 2026-01-12

**Authors:** Ema Dejhalla, Ema Ahel Ledić, Petar Žauhar

**Affiliations:** ^1^ Medical Centre for Occupational Health Rijeka, Rijeka, Croatia; ^2^ Faculty of Medicine, University of Rijeka, Rijeka, Croatia, uniri.hr; ^3^ Clinic of Otorhinolaryngology, Head and Neck Surgery, Clinical Hospital Center Rijeka, Rijeka, Croatia, kbc-rijeka.hr; ^4^ Radiology Department, Clinical Hospital Center Rijeka, Rijeka, Croatia, kbc-rijeka.hr

**Keywords:** child, intracranial, mastoiditis, otitis media, sinus thrombosis

## Abstract

**Introduction:**

Acute mastoiditis, a complication of acute otitis media (AOM), remains a concern in children despite vaccines and antibiotics. Serious complications like cerebral venous sinus thrombosis (CVST) can be life‐threatening.

**Case Presentation:**

A 4‐year‐old girl with fever, vomiting, and ear pain worsened despite amoxicillin treatment, developing headache, neck stiffness, and drowsiness. An ENT examination showed thickened tympanic membranes. Neurological symptoms, including headache, drowsiness, and neck stiffness, prompted lumbar puncture and initiation of ceftriaxone, with elevated CRP and leukocytosis supporting the clinical suspicion. Imaging confirmed otomastoiditis with epidural extension and CVST. MRI confirmed intracranial complications, prompting anticoagulation. She underwent mastoidectomy and tympanostomy tube placement, recovering well.

**Conclusion:**

Early imaging and multidisciplinary management are essential to prevent neurologic complications in children with mastoiditis.

## 1. Introduction

Acute mastoiditis is an inflammatory condition of the mastoid bone, typically arising as a complication of acute otitis media (AOM) [1]. Although its incidence has declined due to widespread vaccination and antibiotic use, it remains a relatively frequent diagnosis in pediatric populations. While most cases follow a benign course, acute mastoiditis can lead to serious intracranial complications, including abscesses, cranial nerve palsies, meningitis, osteomyelitis, and cerebral venous sinus thrombosis (CVST).

CVST is a particularly rare but potentially life‐threatening complication in children with AOM, with significant implications for morbidity due to elevated intracranial pressure (ICP), risk of venous infarction, and long‐term neurodevelopmental consequences [2]. Given its rarity and potential for rapid deterioration, prompt recognition and management are essential—and often challenging.

Clinical presentation can range from classic signs of mastoiditis—such as postauricular swelling and tenderness—to neurological symptoms including headache, lethargy, neck stiffness, and cranial nerve dysfunction. Rare complications, such as pneumocephalus, can further obscure the diagnosis. Magnetic resonance imaging (MRI) and venous angiography are critical for early detection, while laboratory studies typically reveal elevated inflammatory markers [3].

Management begins with intravenous antibiotics, but in more severe cases, anticoagulation therapy may be warranted to prevent progression of thrombosis. Surgical intervention, such as mastoidectomy or thrombectomy, may also be necessary [4]. Diagnosis relies on a clinical history of AOM combined with localized mastoid inflammation. However, prior antibiotic use can mask typical symptoms, potentially delaying diagnosis and allowing complications like CVST to develop unnoticed [5].

## 2. Case Report

A four‐year‐old girl presented with a six‐day history of fever (up to 39°C), respiratory symptoms, and ear pain. Her condition began with nasal congestion and progressed to vomiting after antipyretics, worsening drowsiness, decreased fluid intake, and a rising fever reaching 40°C.

She was born at 36 weeks of gestation via induced vaginal delivery due to maternal hypertension, with a birth weight of 2700 g and normal neonatal adaptation. Perinatal and family histories were unremarkable. She was appropriately vaccinated per the national immunization schedule.

On initial evaluation, she appeared drowsy but responsive, febrile (39°C), pale, and mildly dehydrated. Vital signs included blood pressure of 94/57 mmHg, heart rate of 150 bpm, respiratory rate of 15 breaths per minute, and oxygen saturation of 100% on ambient air. Her weight was 12.5 kg and height 95 cm.

An ENT examination revealed nasal obstruction, hyperemic palatine arches, and postnasal mucopurulent discharge. Tympanic membranes were thickened and opaque, so the initial diagnosis was AOM based on the ENT examination.

Respiratory examination showed normal breath sounds, without wheezing, rales, or respiratory distress.

Cardiac examination revealed rhythmic heart sounds with clear tones and no murmurs.

Neurological examination showed equal, reactive pupils; meningeal signs were absent; and patellar reflexes were preserved bilaterally.

The initial differential diagnosis included viral upper respiratory tract infection, AOM, acute bacterial sinusitis, and—less likely—pneumonia.

She was started on amoxicillin‐clavulanate suspension (80 mg/11.4 mg per mL) at a dose of 3.5 mL twice daily for a presumed bacterial infection. After two days without improvement and with ongoing vomiting, headache, and persistence of symptoms, therapy was switched to cefpodoxime (10 mg/mL), 6 mL twice daily. An ENT evaluation confirmed tympanic membrane changes as the only diagnostic criterion. She was referred for further pediatric assessment, where neck stiffness was noted, prompting hospitalization.

Laboratory investigations revealed:

• CRP: 246.7 mg/L
• Leukocytosis: 17.5 × 10^9^/L
• D‐dimer: 2.07 μg/mL

Due to drowsiness, vomiting, and clinical suspicion of meningitis, a lumbar puncture was performed. Ceftriaxone reconstituted to 100 mg/mL; dose: 600 mg (6 mL) IV, twice daily, for 10 days, was initiated empirically, replacing cefpodoxime. CSF was sterile but showed pleocytosis, and blood cultures grew Acinetobacter ursingii, considered a contaminant.

The patient remained febrile on day one, became subfebrile over the next 2 days, and was afebrile at discharge.

**Figure 1:**
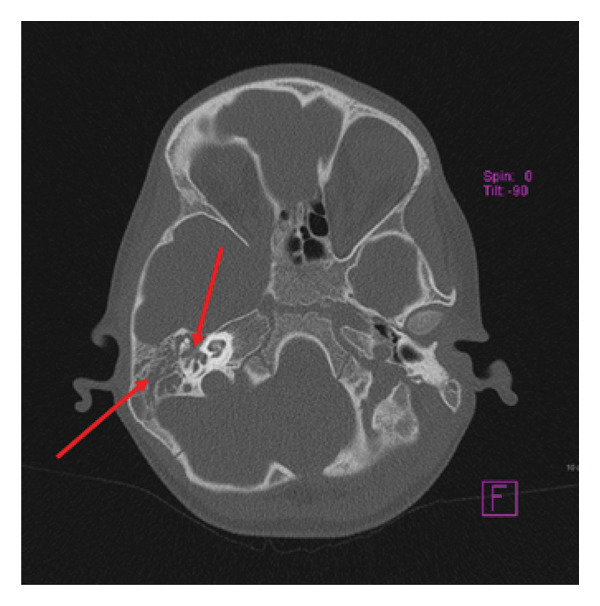
Temporal bone CT‐filled mastoid cells with pathological substrate, thinning and destruction of septa, and involvement of the middle ear cavity (imaging findings).

Brain CT was unremarkable, but temporal bone CT (Figure 1) showed bilaterally filled mastoid cells with septal thinning and destruction, middle ear involvement, retracted tympanic membrane, and possible epidural extension, suggesting otomastoiditis. MRI suggested epidural extension through a focal fluid collection near the right mastoid dura. As there were no neurological deficits or mass effects, the patient was managed conservatively without neurosurgical intervention. MRI with contrast was recommended.

**Figure 2:**
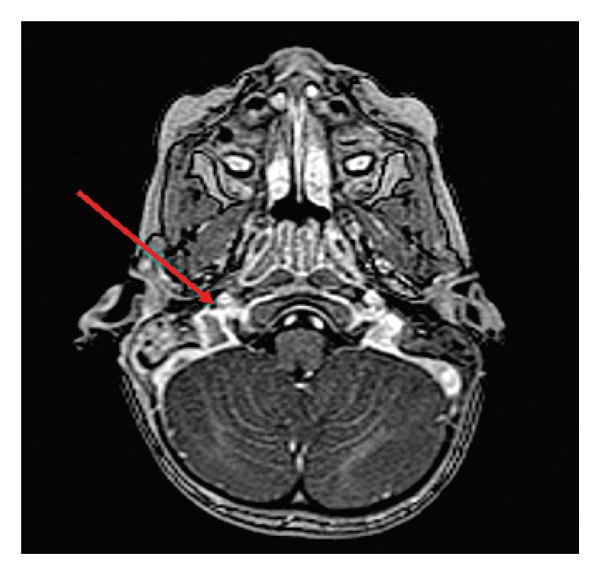
Brain MRI—internal jugular vein and venous sinus thrombosis (right‐sided).

A native and contrast‐enhanced MRI of the brain with angiography was performed. Figure 2 shows a native MRI scan of the brain with angiography. It reveals extensive coalescent mastoiditis on the right side, characterized by a focal lamellar fluid collection (up to 2 mm in thickness) along the posteromedial segment of the mastoid bone base, extending over approximately 15 mm in length. There is a slightly increased signal intensity at the apex of the right petrous temporal bone and focally along the wall of the carotid canal, though without clear MRI criteria for petrous apicitis. The brain parenchyma displays a normal signal pattern, with mildly widened perivascular spaces subcortically in the frontoparietal regions. The ventricular system, sellar region, optic chiasm, cavernous sinuses, and craniocervical junction appear normal, with no signs of acute ischemia, hemorrhage, or neoplastic processes.

**Figure 3:**
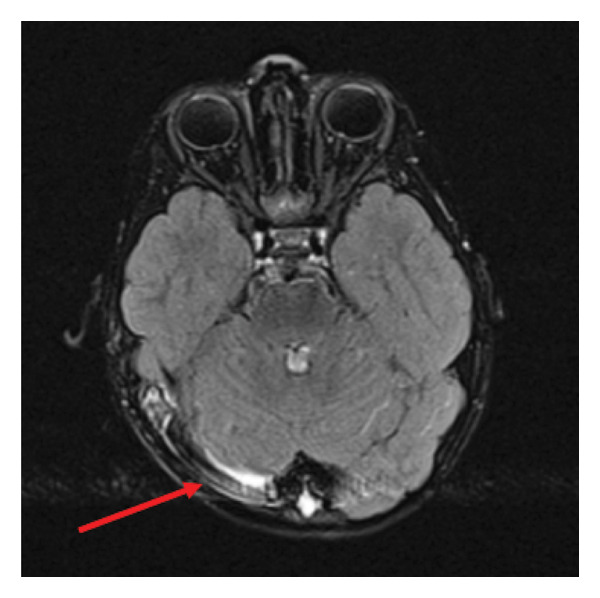
Brain MRI—lamellar collections along the sigmoid sinus (right‐sided).

Figure 3 highlights extensive filling defects in the region of the terminal part of the internal jugular vein, the jugular bulb, and the right sigmoid and transverse sinuses, consistent with thrombosis, with less extensive partial thrombosis on the left side. In contrast‐enhanced FLAIR images, there is an increased signal within individual cerebellar and bilateral occipital sulci, raising the possibility of leptomeningeal inflammation (meningitis), though definitive confirmation is not possible. The gyral and sulcal patterns are appropriate for the patient’s age, and no intraparenchymal spread of inflammation is observed.

Heparin (2 × 10 mg SC) was initiated. ENT specialists recommended mastoidectomy, and the patient was transferred to the otorhinolaryngology department. Further thrombophilia testing (protein C, S, apolipoprotein A, and antiphospholipid antibodies) was normal.

She underwent right‐sided mastoidectomy and tympanostomy tube placement. An intraoperative microbiological swabs identified *Staphylococcus warneri* at low colony‐forming unit (CFU) counts and was isolated from a single specimen only. Given its common status as a skin commensal and the absence of matching growth in other cultures, it was considered a likely contaminant rather than a true pathogen. Postoperatively, she received ceftriaxone reconstituted to 100 mg/mL; dose: 600 mg (6 mL) IV, twice daily as recommended by the infectious disease specialist for seven days, heparin (2 × 10 mg SC), and analgesics. The drain was removed after four days, with no secretion noted. Inflammatory markers normalized (CRP: 2.1 mg/L, leukocytes: 10.6 × 10^9^/L), and D‐dimer was lower (0.77 μg/mL).

She was transferred back to the pediatric department, and rivaroxaban (2 × 5 mg) was introduced on day four. Switching to rivaroxaban allowed for the continuation of anticoagulation therapy in an outpatient setting, which is particularly important for pediatric patients after hospital discharge. Therefore, despite its off‐label status, the decision to transition to rivaroxaban can be clinically justified based on current safety data, practicality, and growing evidence. ENT follow‐up after seven days included suture removal and ceftriaxone completion. At three months, MR venography showed minimal residual thrombus at the jugular bulb, warranting continued anticoagulation. After five months, a brain MRI showed no thrombotic remnants, allowing anticoagulation discontinuation. Beyond radiological improvement and laboratory signs of recovery (CRP: 1.9 mg/L, leukocytes: 9.6 × 10^9^/L, D‐dimer 0.49 μg/mL), the patient showed clear clinical signs of recovery, including resolution of drowsiness and headache, stable vital signs (heart rate of 100 beats per minute, respiratory rate of 25 breaths per minute, blood pressure of 90/55 mmHg, oral temperature of 36.5, and oxygen saturation of 100% on ambient air) and regained oral intake and activity appropriate for age. An immunological diagnostic workup has not been performed during the follow‐up.

## 3. Discussion

Distinguishing between uncomplicated and complicated mastoiditis is essential for appropriate management. In our case, the patient presented with neurological symptoms, including drowsiness and neck stiffness, prompting imaging that revealed CVST and elevated ICP—complications reported in approximately 5%–30% of cases [6]. While isolated lateral sinus thrombosis was more commonly reported in earlier series, most pediatric otogenic CVST today involves multiple venous structures. Multisinus involvement increases the risk of further complications, including mastoiditis progression, intracranial abscesses, and jugular vein thrombosis [6]. Historically, intracranial abscesses were considered the most common complication; however, recent data suggest that venous sinus thrombosis and ICP elevation are now more frequent [1, 2].

Children with mastoiditis complicated by thrombosis often present atypically, with neurological rather than classic otologic symptoms. For example, strabismus may serve as an indicator of sinus thrombosis due to the anatomical proximity of the petrous apex periosteum to the middle ear. This proximity facilitates complications such as pachymeningitis, extradural abscesses, and thrombophlebitis of adjacent venous sinuses [7].

Diagnostic strategies differ significantly between uncomplicated and complicated mastoiditis. While uncomplicated cases often respond well to antibiotics alone and rarely require imaging, fundoscopy and neuroimaging, CT, or MRI with venography are crucial for detecting elevated ICP and thrombosis in complicated cases [8].

The reported prevalence of thrombophilia in mastoiditis‐associated thrombosis varies widely (0%–90%) [9], largely reflecting differences in study populations, diagnostic criteria, and whether genetic or acquired causes were assessed. Nevertheless, genetic thrombophilia accounts for only about 20% of cases, indicating that infection‐driven inflammation and delayed or ineffective treatment play major roles in pathogenesis [10].

In this patient, initial intravenous antibiotic therapy was complemented by anticoagulation, starting with heparin and later transitioning to rivaroxaban. Although rivaroxaban’s use in children remains off‐label, emerging evidence supports its safety and efficacy, offering practical advantages such as oral administration and less frequent monitoring compared to traditional anticoagulants. This approach facilitated outpatient management while maintaining effective thrombosis control [11].

Surgical intervention remains debated and individualized based on disease severity. While mastoidectomy and more conservative procedures like myringotomy are common, extensive surgery or thrombectomy may be required in severe or refractory cases [12]. In our patient, a multidisciplinary approach including pediatricians, infectious disease specialists, radiologists, and neurologists guided timely and appropriate care.

Lastly, given the severity and complications observed, an extended immunological workup would have been beneficial to exclude underlying immunodeficiencies predisposing to recurrent or complicated infections [13]. Early identification of such conditions can influence long‐term management and improve outcomes.

In summary, this case underscores the critical need for heightened clinical suspicion and prompt neuroimaging in children with mastoiditis presenting neurological symptoms, as early diagnosis and combined medical‐surgical treatment are paramount to preventing morbidity from CVST and intracranial complications.

## Consent

The patient has completed and signed the patient consent form.

## Conflicts of Interest

The authors declare no conflicts of interest.

## Funding

No funding was received for this manuscript.

## Data Availability

The data that support the findings of this study are available from the corresponding author upon reasonable request.
